# Human polyomaviruses and cancer: an overview

**DOI:** 10.6061/clinics/2018/e558s

**Published:** 2018-09-26

**Authors:** José Carlos Mann Prado, Telma Alves Monezi, Aline Teixeira Amorim, Vanesca Lino, Andressa Paladino, Enrique Boccardo

**Affiliations:** Departamento de Microbiologia, Instituto de Ciencias Biomedicas, Universidade de Sao Paulo, Sao Paulo, SP, BR

**Keywords:** MCPyV, BKPyV, JCPyV, TSPyV, Human cancer

## Abstract

The name of the family *Polyomaviridae*, derives from the early observation that cells infected with murine polyomavirus induced multiple (*poly*) tumors (*omas*) in immunocompromised mice. Subsequent studies showed that many members of this family exhibit the capacity of mediating cell transformation and tumorigenesis in different experimental models. The transformation process mediated by these viruses is driven by viral pleiotropic regulatory proteins called T (tumor) antigens. Similar to other viral oncoproteins T antigens target cellular regulatory factors to favor cell proliferation, immune evasion and downregulation of apoptosis. The first two human polyomaviruses were isolated over 45 years ago. However, recent advances in the DNA sequencing technologies led to the rapid identification of additional twelve new polyomaviruses in different human samples. Many of these viruses establish chronic infections and have been associated with conditions in immunosuppressed individuals, particularly in organ transplant recipients. This has been associated to viral reactivation due to the immunosuppressant therapy applied to these patients. Four polyomaviruses namely, Merkel cell polyomavirus (MCPyV), Trichodysplasia spinulosa polyomavirus (TSPyV), John Cunningham Polyomavirus (JCPyV) and BK polyomavirus (BKPyV) have been associated with the development of specific malignant tumors. However, present evidence only supports the role of MCPyV as a carcinogen to humans. In the present review we present a summarized discussion on the current knowledge concerning the role of MCPyV, TSPyV, JCPyV and BKPyV in human cancers.

## INTRODUCTION

Polyomaviruses (PyVs) are icosahedral, nonenveloped viruses that are approximately 45 nm in size. The capsid is composed of 72 pentameric capsomers and surrounds a circular, double-stranded viral DNA genome that is approximately 5,5 kbp. Inside the virion, the DNA is associated with the cellular histones H2A, H2B, H3 and H4, forming the viral minichromosome. In addition, inside the cell, the minichromosome is found to be associated with histone H1 1. These viruses are relatively resistant to treatment with formalin and are not affected by organic solvents 2.

Currently, the International Committee on Taxonomy of Viruses (ICTV) has divided the *Polyomaviridae* family into 5 genera: *Alphapolyomavirus,* with 37 species that infect animals and humans, including human PyVs (HPyVs) 5, 8, 9, 12 and 13; *Betapolyomavirus*, with 29 species that infect animals and humans, including HPyVs 1 and 4; *Deltapolyomavirus* with only HPyVs 6, 7, 10 and 11; *Gammapolyomavirus*, with seven animal virus species, including PyVs that infect birds; and, finally, an unassigned genus that contain three species of animal PyVs 3,4.

### Biology of polyomaviruses

The genomes of PyVs can be divided into three regions: the control region; the early region, which encodes early proteins; and the late region, which encodes late proteins with structural function. The control region contains the origin of replication and the promoters that regulate the expression of early and late genes. However, this region does not encode any protein or functional RNA. This region is involved in the regulation of the viral life cycle by modulating replication and transcription. Both strands of the PyV DNA code for proteins. Early genes are expressed from one strand immediately after infection. On the other hand, late genes are expressed from the opposite strand after viral genome replication. The mRNA transcribed from both early and late regions exist in at least two isoforms due to alternative processing. In addition, the expression of a viral microRNA has been observed in the John Cunningham polyomavirus (JCPyV), BK polyomavirus (BKPyV) and Merkel cell polyomavirus (MCPyV) 5,6. This microRNA is encoded by the DNA strand complementary to the large T antigen (see below) and, at least in MCPyV, this RNA seems to regulate the expression of this viral protein 5.

The proteins encoded by the early genes are involved in the regulation of viral transcription and genome replication. These proteins are known as “T antigens” and received this name because they were recognized by antibodies from rodents bearing tumors 7. Different T antigen isoforms exist and are named depending on the viral species from which they originate. The transforming potential of the viral group is directly related to the expression of T antigens and was initially established in studies using simian virus 40 (SV40) 8. The large T (LT) antigen is a nuclear protein that is approximately 700 amino acids. However, alterations in its phosphorylation pattern may change the location of this protein within the cell 9,10. The LT antigen regulates both viral transcription and genome replication 11. In addition, the LT antigen as well as the other T antigen isoforms expressed in human and animal PyVs are pleiotropic proteins that affect the function and expression of several cellular proteins involved in the regulation of cell proliferation (see below). The neutralization of the functions of these proteins is critical for the induction of the entry of the host cell into the cell cycle, making the DNA replication machinery available and allowing viral genome replication 12-15.

The late region harbors the genes that encode structural proteins found in the viral capsid, such as VP1 and VP2. Some species also express the structural proteins VP3 and VP4. VP2 and VP3 are important for viral entry into the host cell. However, the role of VP4 during the viral life cycle remains a matter of debate. Previous studies have suggested that VP4 acts as a viroporin to disrupt the nuclear envelope and mediate viral release 16-19. However, in a recent study, Henriksen et al. showed that human renal proximal tubule epithelial cells transfected with BKPyV genomes carrying start codon substitutions in VP4, predicted to abolish the production of this protein, released comparable amounts of viral particles in the supernatant as cells transfected with WT genomes 20. It is clear that additional studies are needed to determine the role of VP4 in PyV biology. In addition, some PyVs, including JCPyV, BKPyV and SV40, present an open reading frame (ORF) that encodes a regulatory cytoplasmic protein named agnoprotein 21. The involvement of agnoprotein in viral release has been suggested in species that express this protein, including SV40, JCPyV and BKPyV 22.

Studies conducted during the past two decades have shown that the expression of VP1 in different systems leads to the production of structures called virus-like particles (VLPs), which are similar to the viral capsid 23-27. After being expressed, the structural proteins accumulate in the cellular nucleus, contributing to the mounting of the virion. These proteins are found in different amounts in viral capsids, with VP1 being the major protein in the formation of pentamers. The carboxy end of VP1 extends outside the pentamer and interacts with surrounding pentamers. These interactions, mediated by VP1 together with Ca^2+^ and disulfide bonds, contribute to the stabilization of the capsomers and capsid structure 2.

The mechanism of viral entry into the host cell seems to depend on the virus and cell type under study. For instance, cell internalization via caveolin- or clathrin-dependent endocytosis, as well as entry via other mechanisms, has been previously described 28,29. Once in the cytoplasm, the viral capsid suffers alterations that expose the nuclear localization signals present in proteins VP2 and VP3 and mediate nuclear import. After reaching the nucleus, the capsid is completely dismounted, and the viral genome is exposed and maintained in the episomal form. Next, the early region is transcribed to produce an mRNA molecule that, after processing, will generate the large T antigen and its other isoforms. The LT antigen protein will first mediate viral genome replication. This event may also be regulated by epigenetic mechanisms due to the association of viral DNA with cellular histones 30. Only after genome amplification will the LT antigen promote the transcription of the late region to express the structural proteins. Infective virions are mounted in the nucleus, and the release of these virions may depend on the occurrence of cell lysis 2,31.

The majority of PyV infections are asymptomatic; however, these infections may cause alterations in cell cultures and induce tumors in immunocompromised laboratory animals, including newborn mice. The transforming potential of PyVs has been demonstrated *in vitro* using cells from different organisms 32. Early studies conducted in immunocompromised animals injected with murine polyomavirus (MuPyV)-infected cells showed the formation of multiple (*poly*) tumors (*omas*) and served to name the group 7.

### Human polyomaviruses

**Figure 1:**
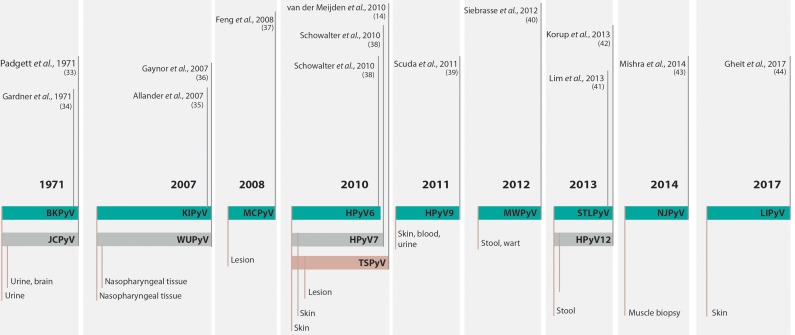
Timeline of the discovery of human polyomaviruses. The timeline shows the name of the virus, the year of discovery and the type of sample from which the virus was isolated. For complete references, see the text. BKV or BKPyV, human polyomavirus BK or human polyomavirus 1; JCV or JCPyV, John Cunningham or JC polyomavirus or human polyomavirus 2; KIPyV, Karolinska Institute polyomavirus or human polyomavirus 3; WUPyV, Washington University polyomavirus or human polyomavirus 4; MCPyV, Merkel cell polyomavirus or human polyomavirus 5; HPyV6, human polyomavirus 6; HPyV7, human polyomavirus 7; TSPyV, Trichodysplasia spinulosa polyomavirus or human polyomavirus 8; HPyV9, human polyomavirus 9; MWPyV, Malawi polyomavirus or human polyomavirus 10; STLPyV Saint Louis polyomavirus or human polyomavirus 11; HPyV12, human polyomavirus 12; NJPyV, New Jersey polyomavirus or human polyomavirus 13; LIPyV, Lyon IARC polyomavirus or human polyomavirus 14.

The discovery and characterization of HPyVs occurred in different technological contexts. In 1971, two isolates were described, and for decades, these species remained the only members of the family that infected humans 33,34. It was not until 2007, and only after major advances in DNA sequencing technologies, that other twelve PyVs were identified in human samples, increasing the total number of HPyVs to fourteen 14,. The timeline of HPyV discovery is summarized in Figure 1.

Serological studies have shown that exposure to most HPyVs occurs early in life and that infection prevalence in adults may be high 45. Interestingly, immunocompromised individuals exhibit higher viral loads of these agents suggesting that immunocompromised individuals are more susceptible to reactivation of these viruses 14,46-49. Among the PyVs that affect humans and are associated with important diseases in immunocompromised individuals, the most relevant are JCPyV, BKPyV, MCPyV and Trichodysplasia spinulosa PyV (TSPyV). These PyVs are ubiquitous and are highly prevalent in the normal human population. In general, infection by these agents is asymptomatic in healthy individuals, with prevalence values as high as 80% in adults, as determined by serological studies 27,50. On the other hand, in immunocompromised persons, infection with BKPyV is associated with the development of nephropathies and hemorrhagic cystitis, while infection with JCPyV is associated with progressive multifocal leukoencephalopathy (PML). In addition, MCPyV is associated with the development of Merkel cell carcinoma (MCC) and is considered a group 2A carcinogen (probably carcinogenic to humans) by the International Agency for Research on Cancer 51.

It is accepted that PyV-associated tumors develop after the interruption of the viral life cycle. This may be caused by accidental viral integration into de cellular genome. In the case of MCPyV and BKPyV, viral cycle interruption may be caused by rupture of the VP1 gene 52,53 or by loss of the carboxy-terminal domain of the LT antigen due to nonsense mutations. This domain of the LT antigen is needed for the normal cycle to promote viral genome replication 52,37. Therefore, viral replication is interrupted while truncated versions of the LT antigen and its isoforms are expressed 52.

The presence of the LT antigen is a universal characteristic of the members of the *Polyomaviridae* family. LT antigens share high identity between the members of the PyV genus, which allows recognition by cross-hybridization in western blots performed with a few specific antibodies 54. The N-terminal region of this protein contains a DnaJ domain, which contributes to viral replication and mediates the binding of the cellular chaperone HSc70. This protein also contains an LXCXE motif that binds the members of the retinoblastoma protein family pRb, p107 and p130. Together DnaJ and LXCXE disturb the pRb/E2F complexes, promoting the progression of the cell cycle 11. The C-terminal domain of the LT antigen harbors a conserved threonine residue that, when phosphorylated, competes with cyclin E1 and Myc to bind to FBXW7. The protein FBXW7 (F-box) is part of the ubiquitin ligase complex formed by Skp1/culin/F-box (SCF). As such, LT prevents the degradation of cyclin E1 and Myc and contributes to cell growth and proliferation 31.

The LT antigen also presents a nuclear localization sequence (NLS), an origin-binding domain (OBD) and a helicase domain. The OBD and the helicase domain are critical for viral genome replication 55. Finally, LT antigens from many, but not all, HPyVs exhibit a p53-binding domain 56,57. The colocalization of p53 and the LT antigen has been demonstrated in the cytoplasm of cultured cells of BKPyV-positive neuroblastoma and prostate cancer 58,59. It is believed that this interaction may block the expression of p53-regulated genes in response to DNA damage 57. Another cellular target of early PyV proteins is the phosphatase PP2A 60. This protein is inactivated by T antigens from SV40, JCPyV and MCPyV 59. PP2A is a critical regulator of the mitogen-activated protein kinase (MAPK) signaling cascade and has many functions. This protein contributes to the control of cellular metabolism by regulating the activities of different enzymes involved in glycolysis, lipid metabolism and catecholamine synthesis 61. In addition, this protein regulates various critical processes, such as cell cycle progression, DNA replication and transcription, protein translation, signal transduction, cytoskeletal dynamics, cell mobility and apoptosis. As such, PP2A plays an important role in cell transformation and cancer 62-65.

Most HPyVs have the potential to cause nonneoplastic diseases in the context of immunosuppression 54. However, this association has only been confirmed for a few of these viruses. Among the 4 PyVs associated with diseases in immunosuppressed humans, two are associated with proliferative diseases of the skin. MCPyV is associated with MCC, while TSPyV is the etiological factor for Trichodysplasia spinulosa (TS). The two other viruses are JCPyV, which is associated with PML, and BKPyV, which is a leading cause of chronic dysfunction in renal transplant patients, urethral stenosis and nephropathy. The main characteristics of these viruses will be presented in the next sections. Moreover, the cell transforming potential exhibited by different HPyV proteins *in vitro* raises the intriguing possibility that some of these agents, in addition to MCPyV, may be associated with specific human cancers. Therefore, several studies conducted by different groups around the world have addressed the presence of all HPyVs in different human tumors. Table 1 presents a comprehensive summary of the main results obtained.

### Merkel cell polyomavirus and Merkel cell carcinoma

In 2008, the identification of the fifth HPyV, detected in samples of MCC, was reported, and this virus was named MCPyV. The authors performed digital transcriptome subtraction (DTS) in MCC samples and identified one sequence that exhibited no similarity with human transcripts. In-depth analysis of the transcript using nucleotide databases showed that this sequence was related to the T antigen sequence from simian lymphotropic polyomavirus (LPyV) and BKPyV. The viral genome, which was detected in 80% of the MCC samples, was amplified by *primer walking* and sequenced. The study also showed that in most of the MCPyV-positive tumors, the viral DNA exhibited a clonal integration pattern within the cell genome. The same integration pattern detected in primary tumors was observed in the derived metastases, supporting the notion that viral integration preceded the clonal expansion of tumor 37.

MCC, a rare and aggressive neoplasia, was described in 1972 by Toker as a trabecular carcinoma of the skin 66. Data from the Netherlands show an incidence rate of 0,35 cases per 100.000 per year (0,35/100.000), while the incidence in the USA is 0,24/100.000 67,68. On the other hand, in Queensland (Australia), where most habitants are Caucasian, the incidence increases to 1,6/100.000 69. This tumor is more prevalent in men (61% of the cases) than in women, particularly in white individuals older than 65 years 68-70. This pathology is well described by the acronym AEIOU: “Asymptomatic/lack of tenderness, Expanding rapidly, Immune suppression, Older than age 50, and UV-exposed site on a person with fair skin” 71. MCC occurs as fast-growing reddish-blue nodules located mainly over soft tissues, sometimes with telangiectasia, in areas exposed to intense solar radiation. The occurrence of MCC varies from 41% to 50% in the head and neck, 32% to 38% in the limbs and 12% to 14% in the trunk of the body 72. In addition, MCC can be detected in anatomic areas with low UV exposure, such as genitalia and mucosa 71. Interestingly, a retrospective study conducted in the USA showed that MCC cases in black people occur mainly at the extremities of the inferior limbs 73. The ultimate diagnosis of MCC is given by histopathological analysis of biopsies. This tumor presents small ovoid cells with hyperchromatic nuclei, which are characteristic of neuroendocrine tumors. The tumor architecture may be trabecular, nodular or diffuse 74. The best immunohistochemical markers for this pathology are neurofilaments and cytokeratin 20 75.

#### Merkel cell polyomavirus biology and epidemiology

**Figure 2:**
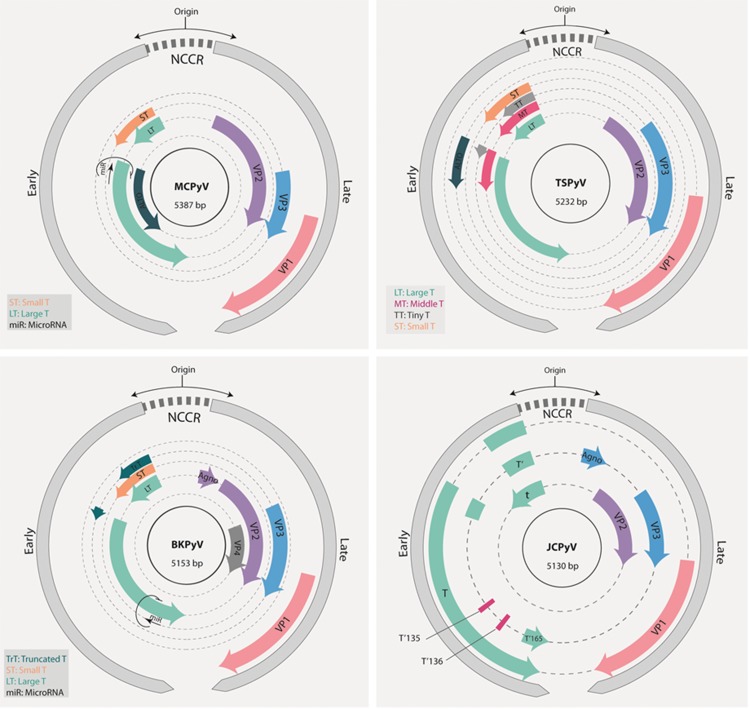
Schematic representation of the genomes of BKPyV, JCK, MCPyV and TSPvV. The early and late regions (gray) are transcribed from opposite strands of the genome. The early region is transcribed in the counterclockwise direction and harbors the genes coding for the different T antigen isoforms as indicated. The late region expresses the structural genes (VPs) and the agnoprotein ORF (when present). BKPyV, JCV and MCPyV express a microRNA from the opposite strand of the early region. The noncoding control region (NCCR) contains the origin of genome replication and the promoters for the regulation of transcription. For details, see text.

The MCPyV genome comprises 5387 bp (isolate MCC350, EU375803) and exhibits the characteristic organization of the family (Figure 2). The early region of this virus expresses the large T (LT), small (sT), and 57kT antigens 76. In addition, in 2013, the existence of a fourth ORF, expressed from an alternative transcription initiation site located in the second exon of the gene coding for the LT antigen was described. The protein coded by this gene is called alternate frame of the large T ORF (ALTO); this protein is expressed in replicating infected cells and remains in the cytosol. The role of this protein in the viral cycle and associated diseases has not been determined. Intriguingly, the ORF that encodes this protein has been found to be mutated in tumor tissues, suggesting that this protein may play a role in viral pathogenesis 77. The importance of the LT antigen in the pathology mediated by this virus is highlighted by the fact that silencing of this antigen in MCPyV-positive MCC-derived cells inhibits cell growth and induces senescence 78. Several studies have demonstrated that MCPyV genomes present in tumors exhibit mutations in the 3' region of the gene encoding the LT antigen, mainly at the region upstream of the helicase domain and downstream of the gene encoding the sT antigen 53,79-81. The accumulation of mutations in this portion of the LT antigen is important in the process of carcinogenesis since these mutations downregulate viral replication and viral load, allowing immune evasion while retaining the ability to promote unscheduled cell proliferation. This phenomenon is possible because the mutated form of the LT antigen always preserves the domains that are involved in interactions with cellular factors, including the domain that targets pRb 82. The sT antigen, a 186-amino-acid protein, harbors the site for PP2A binding. This site is conserved among PyVs and plays a role in cellular transformation 60. In addition, the sT antigen promotes LT-antigen-dependent MCV genome replication by sequestering the F-box and WD repeat domain containing 7 (FBXW7) component of the Skp, Cullin, F-box (SCF)-containing ubiquitin ligase responsible for LT antigen degradation by the proteasome 76,83. The amino acids at position 91 to 95 of the sT antigen are required for this function and define the large T stabilization domain (LSD). Mutation of the LSD in the sT antigen leads to downregulation of the LT antigen and prevents viral genome replication. Mutations in this domain also prevent rodent cell transformation and induction of cellular oncoproteins, including c-Myc and cyclin E, by the sT antigen 84. Moreover, *in vitro* and *in vivo* observations indicate that LSD integrity is required for sT mediated induction of supernumerary centrosomes, appearance of aneuploid cells, accumulation of chromosomal breaks and micronuclei 85. Importantly, sustained expression of the sT antigen in MCPyV is required for tumor cell proliferation, which has been linked to sT-antigen-mediated stabilization of the eukaryotic translation initiation factor 4E-binding protein 1 (4E-BP1), which leads to increased cap-dependent translation in infected cells 86. Moreover, a recent study conducted using an *in vivo* model of MCC showed that expression of the sT antigen with an intact LSD domain was critical for tumor initiation. On the other hand, coexpression of LT did not affect the frequency of tumor establishment 87. Finally, the MCPyV sT antigen is more frequently detected in human MCC tumors than the LT antigen. These observations suggest a critical role for the sT antigen in MCPyV-mediated carcinogenesis. As previously mentioned, MCPyV integration into the genome of the host cell, which interrupts normal viral cycle regulation, is a critical step in MCC development 76. To date, there has been no report of MCCs harboring MCPyV DNA in the episomal state. Importantly, a truncated form of the LT antigen or its complete smaller isoforms continue to be expressed, altering cell homeostasis 88-91.

Finally, the MCPyV early region expresses a microRNA with no identified cellular target but complementary to the 3' portion of the LT antigen, suggesting the involvement of this microRNA in the regulation of the expression of the viral protein 5.

The late region harbors the genes that encode the structural proteins VP1, VP2 and VP3. Interestingly, VP3 is not detected in MCPyV-infected cells or in MCPyV virions. Moreover, alteration of the initiation codon of the VP3 ORF does not alter the infectivity of MCPyV in cell culture. These observations indicate that VP3 may be expressed under only certain conditions 92.

The study of MCPyV prevalence in the human population suggests that this virus is part the skin microbiota 38. Exposure to this agent occurs early in life, as demonstrated by serological surveys, which showed that 20% to 40% of children less than five years old test positive for antibodies against this virus. In addition, positivity increases to 80% in individuals more than 50 years old 27,50,93-97. Transmission may occur via direct contact with the skin or saliva 98. In addition, airborne as well as fecal-oral routes of transmission have been proposed 99-101. A prospective study conducted with bisexual and homosexual adults who were controlled at six month intervals showed that primary infection is asymptomatic in most of the cases. Analysis of clinical variables such as fever, presence of sprouts, diarrhea or loss of weight, as well as cytological tests involving the counting of erythrocytes and lymphocytes (including CD4 and CD8 populations) were unable to differentiate control individuals from those that had seroconverted 97. As described above, MCPyV is considered to be a part of the skin microbiota. However, detection of viral DNA is very frequent in patients with MCC, even at sites that are distant from the lesion 80,102. Viral DNA has been detected in blood, eyebrows, nasal swabs and aspirates, and adrenal glands 80,99,101-104. The presence of this virus has been analyzed in other tissues and was not detected in samples from the central nervous system 105. However, analysis of viral presence in lymphoid tissues led to unconclusive results 106-108.

#### Pathogenesis of Merkel cell polyomavirus

Merkel cells are located at the basal layer of the epithelia of the skin and oral mucosa and are in direct contact with the tactile neural discs, to which these cells transmit mechanical information. Merkel cells are associated with afferent demyelinated neurons of the dermis 109. In the skin, these cells are part of the somatic sensorial system and are classified as exteroreceptors. However, in the epithelium of the mouth, the format and function of these cells increase in complexity 110. Although MCC is diagnosed by the detection of specific markers, namely, cytokeratin 20 and CD56, the origin of Merkel cells remains a matter of debate 111. Considering the analysis of the expression of different cellular markers, several hypotheses have been proposed, including epithelial stem cells and pre-pro-B lymphocytes being the precursors of Merkel cells 112-116. However, independent of the anatomic locations of these cells, when observed by electron microscopy, Merkel cell tumors exhibit neuroendocrine granules, suggesting the neuroendocrine origin of these malignancies 117. Results from a recent study suggest that dermal fibroblasts may be the primary cells infected by MCPyV 118. Interestingly, analysis of MCPyV expression and replication in dermal fibroblasts from different species demostrated that only human and chimpanzee cells were permissive for the production of infectious MCPyV 119. However, no viral DNA or protein has been detected in the dermis adjacent to MCPyV-positive MCCs 120.

Immunosuppression is another important factor in MCC development and progression. In fact, immunocompromised individuals exhibit a higher (16-fold) relative risk for MCC than the normal population 70. Immunosuppression allows the establishment of persistent infections, even in the presence of immunogenic viral antigens 121. In addition, similar to other tumor viruses, including human papillomaviruses, Kaposi's sarcoma herpesvirus and human T lymphotropic virus type-I, MCPyV exhibits a series of immune evasion mechanisms. For instance, it has been observed that the LT antigen inhibits the activity of the transcription factor C/EBPβ, which downregulates the expression of Toll-like receptor 9 122. This fact makes the cell unable to detect unmethylated double-stranded DNA in its cytoplasm 123. Moreover, the sT antigen binds NF-κB (NEMO/IKK-γ), altering an important pathway involved in innate immunity 124. In addition, MCPyV-positive MCCs present lower levels of MHC I than MCPyV-negative tumors. Usually, cells lacking MHC I expression are eliminated by natural killers (NK) cells. Importantly, MCPyV reduces the expression of the NK-activating receptor group 2, member D (NKG2D), allowing the survival of tumor cells expressing low levels of MHC I 125. However, MHC I expression may be induced in MCPyV-positive cells in response to IFN-γ, which may prove relevant for MCC treatment 126.

Studies conducted in different populations have shown that patients diagnosed with MCC are at higher risk of developing second neoplasias 66,127-129. Among these neoplasias, malignant skin tumors are the most frequent, highlighting the effect of UV radiation in the genesis of these different types of tumors 127,130. In addition, lymphoid leukemia is also common in MCC patients 131. Importantly, in many cases, the second neoplasia is an independent primary MCC 132-138.

Finally, the presence of MCPyV has been analyzed in different human tumors. The results from several studies addressing this issue are summarized in Table 1.

### John Cunningham polyomavirus

The human polyomavirus JCPyV is genetically related to BKPyV and SV40. The first report of this virus was made by Zurhein and Chou more than fifty years ago 139. Using electron microscopy, these authors observed the presence of particles similar to papovavirus in oligodendrocytes present in demyelinated areas of the brains of patients with PML 139. The virus was then isolated after the inoculation of brain extracts from a patient with PML (patient John Cunningham) in primary human fetal glial cells 33.

Different studies have shown that 40-60% of all adults exhibit IgG antibodies against the JCPyV VP1 protein 31,51. Initial subclinical infections occur during childhood, and the virus establishes lifelong infections in specific sites, such as the proximal kidney tubule. On rare occasions, the virus may be reactivated. This phenomenon is more frequent in immunosuppressed individuals, such as patients with AIDS or recipients of organ transplants, than in non-immunocompromised individuals 31. Viral reactivation may lead to the development of PML, a fatal demyelinating disorder of the central nervous system caused by the destruction of oligodendrocytes as a consequence of the lytic viral cycle 11,140. In addition, this virus has been associated with renal diseases in immunosuppressed individuals and organ transplant recipients 141-144.

#### Viral biology and epidemiology

The 5130-bp genome of JCPyV (J02226) presents the same general characteristics that are typical of PyVs, as described above 1 (Figure 2). This virus expresses three structural proteins, namely, VP1, VP2 and VP3, from its late region, with VP1 being the major capsid protein 145. In addition, this virus expresses three regulatory proteins. The LT and sT antigens are expressed from the early region, while the gene coding for agnoprotein is located in the late region. Three splicing variants have been reported for the LT antigen, namely, T′135, T′136, and T′_165_, which are expressed in infected cells during the lytic cycle 146,147. In addition, JCPyV expresses a microRNA that may be involved in regulation of the LT antigen, as reported for MCPyV 5.

The JCPyV LT antigen shares structural and functional homology with LT antigens from other PyVs. Similar to LT antigens from other PyVs the JCPyV LT antigen and its splicing variants are multifunctional proteins that interact with viral and host DNA and proteins affecting their functions. As previously mentioned, LT antigens are important for the induction of DNA replication in infected cells, allowing the virus to usurp the DNA replication machinery to amplify its genome 10,11. This occurrence fosters viral multiplication in permissive cells and viral transmission. However, JCPyV infection of nonpermissive cells may lead to cellular transformation 57. Expression of the LT antigen may be regulated by the expression of a viral microRNA complementary to the 3' region of the early mRNA. In addition, this microRNA targets the cellular mRNA that expresses UL16-binding protein 3 (ULBP3) 6, probably leading to inhibition of the antiviral response of NK cells 148.

Other viral proteins are involved in the control of the cell cycle and viral replication. JCPyV also expresses an sT antigen. As described above for TSPyV and JCPyV, the sT antigen binds the phosphatase PP2A, promoting cell proliferation 149. In addition, sT targets members of the pRb family, further affecting cell cycle control 150. The JCPyV agnoprotein has been described as a multifunctional factor 151. Functional elimination of this protein by deletion or mutation leads to a dramatic downregulation of viral genome replication and transcription 22. However, the effect of this protein on host cell homeostasis is not clearly understood. The agnoprotein of JCPyV may bind several cellular factors, including p53, YB-1, Ku70, FEZ1, HP1α, PP2A, AP-3, PCNA, and α-SNAP. In addition, this protein can bind LT, sT and VP1 and regulate the viral cycle 22,152. JCPyV variants carrying deletions in the gene coding for the agnoprotein have been linked to the development of severe encephalopathy, which is of clinical relevance 153.

JCPyV inoculation in animal models, including rodents and nonhuman primates, that are not permissive to the replication of this virus resulted in the formation of tumors. Since then, the tumorigenic potential of this virus in humans and the association of this virus with the development of some human malignancies has been a matter of debate 154 (Table 1). To date, no conclusive prospective studies supporting a causal association between JCPyV infection and cancer development in humans have been conducted. Several case-control studies, sometimes nested within cohort studies, have been conducted to establish the association between JCPyV seropositivity and specific human tumor types, including colorectal cancer 155-161, lymphoma 162,163, central nervous system tumors 164,165-166, esophageal carcinoma 167, carcinoma of the bladder 168, and prostate cancer 169. These results should be interpreted with caution since anti-JCPyV antibodies may persist for decades, indicating previous exposure to the agent but not viral reactivation. On the other hand, detection of JCPyV by qPCR in the urine indicates active replication and has been applied for the detection of the virus in cases of colorectal and bladder carcinomas 170,171.

To date, the oncogenic potential of this virus has not been clearly established. Moreover, although JCPyV DNA has been detected in a varying percentage of gastrointestinal tumors, the IARC classifies this virus as a group 2B carcinogen, indicating that JCPV is possibly carcinogenic to humans 51,161.

It is well established that immunosuppressed individuals exhibit an increased risk of cancer. Few studies have addressed the prevalence of JCPyV in tumors of immunosuppressed patients. In a recent study, Bolting et al. observed a higher prevalence of JCPyV DNA in the normal mucosa of the gastrointestinal tracts of patients who received immunosuppressant therapy than in immunocompetent control individuals (23,7% vs. 6,3%; p = 0,02) 172. Importantly, organ transplant recipients exhibited a relative risk of 10.4 (prevalence 35,3%) for carrying viral DNA. Altogether, these results suggest that persistent viral infection in immunosuppressed individuals may be a risk factor for tumor development. Further studies are needed to confirm this hypothesis. Another study compared the prevalence of JCPyV between the normal colonic epithelium and adenomatous polyps from liver transplant recipients (LTRs) and normal and adenoma tissue samples from control patients 173. The authors observed that LTRs exhibited higher prevalence of JCPyV DNA in the normal colonic mucosa than the control patients (67% vs. 24%, p = 0.025). In addition, the JCPyV LT antigen protein was detected at a higher proportion in adenomas from LTRs than in those from immunocompetent patients (50% vs. 5%, p < 0.001) 173. These results suggest that JCPyV may be reactivated under immunosuppressive conditions.

Altogether, the data discussed above underscore the need for further molecular and epidemiological studies to gain insights into the mechanisms of JCPyV pathogenesis. Molecular studies conducted to better characterize the impact of viral proteins in cellular processes will be needed to determine the mechanisms of JCPyV-mediated cell transformation. In addition, epidemiological, prospective and multicentric studies will be necessary to determine the existence of causality between JCPyV and specific human cancers.

### BK polyomavirus

Ninety percent of adults worldwide have been exposed to BKPyV. As is the case for the other PyVs discussed in this review, initial BKPyV infection occurs during childhood 174. The virus seems to be transmitted via multiple routes, including respiratory, urine-oral, feco-oral, and transplacental and via transplantation of infected organs 174-179.

Four BKPyV genotypes, namely, I, II, III and IV have been described based on variations in the nucleotide sequence of the gene coding the structural protein VP1 and constitute different serotypes 49,180 (Figure 2). BKPyV expresses two minor structural proteins, namely, VP2 and VP3, which participate in nuclear entry upon infection and virion mounting 181. In addition, BKPyV expresses a microRNA with similar functional characteristics as those described for JCPyV 5,6,148. Finally, the BKPyV late region expresses an agnoprotein important for the productive life cycle of the virus 182,183. Agnoprotein has been shown to interact with the N-ethylmaleimide-sensitive factor attachment protein alpha (α-SNAP), affecting the secretion of fusion reporter proteins and supporting a role for this viral protein in the regulation of exocytosis 184. In addition, agnoprotein also targets the proliferating cell nuclear antigen (PCNA), downregulating DNA synthesis and cell proliferation *in vitro* 185. This observation suggests that agnoprotein may inhibit viral DNA synthesis at late stages of the viral cycle to allow virion mounting. The early region of the BKPyV genome expresses LT and sT antigens. Recently, the expression of a truncated form of the T antigen (trT) has been reported 186.

After primary infection, the virus may establish persistent infection in uroepithelial cells, oligodendrocytes and mononuclear cells from the blood 49,174. In a great majority of the cases, the infection is asymptomatic, and the virus can be detected in the urine of 0,5% to 20% of the healthy population 178,187. However, BKPyV can be reactivated in organ transplant recipients due to the immunosuppressant therapy used in these patients. Viral reactivation is associated with increased viral load and destruction of infected tissues 49. In fact, BKPyV is one of the leading causes of kidney transplant failure, and the prevalence of this virus in kidney transplant recipients is high (10% to 60%). In addition, this virus is associated with ureteric stenosis and nephropathy 178,179. Viral reactivation has also been associated with old age, pregnancy, diabetes mellitus, congenic immunodeficiency and AIDS 179. Finally, BKPyV has also been detected in HIV-associated salivary gland disease (HIVSGD), suggesting that this virus may also exhibit tropism for this organ 188.

As observed for other PyVs, the complete BKPyV genome or fragments containing the early genes are able to transform several cell types from different animals in cell culture systems. However, transformation of human cells by BKPyV is inefficient 189. The LT antigen seems to be the main transformation-associated protein in BKPyV. This protein transforms rodent cells and immortalizes human cells in the presence of activated oncogenes such *ras* and *myc* 189,190. *In vitro* studies have shown that the LT antigen from BKPyV binds p53 as well as members of the pRb family 183.

BKPyV DNA has been detected in several human cancers, including carcinomas of the lung, pancreas, liver, urogenital tract, head and neck 191,192. In addition, this virus has been detected in rhabdomyosarcoma, Kaposi's sarcoma and brain tumors 192. The results from these and other studies are presented in Table 1. In addition, a recent study showed that patients with bladder cancer exhibit higher median antibody titers against this virus than matched controls. Moreover, it was observed that the risk of bladder cancer was significantly increased in individuals exhibiting high antibody titers against BKPyV and MCPyV 168.

As in the case of JCPyV, BKPyV is considered a possible human carcinogen (2B) by the IARC. Therefore, comprehensive prospective epidemiological studies are needed in order to prove or refute the role of BKPyV in human cancers.

### Concluding remarks

The proven cell transforming ability of different members of the *Polyomaviridae* family has promoted to studies to determine the existence of an association between different HPyVs and cancer development. The first HPyVs were described more than 45 years ago. However, recent technological advances have led to the swift identification of several PyVs in human samples. Epidemiological and molecular data has established MCPyV as a *bona fide* tumor virus. Moreover, BKPyV and JCPyV are possibly involved in the etiology of specific human malignancies.

In fact, there is a possibility that other HPyVs may be related with different malignancies in humans. Multiple studies conducted during the last decade have addressed the presence of almost all the known HPyVs in human tumors (Table 1). For instance, TS lesions associated with TSPyV infection that affect individuals who receive immunosuppressing therapies are characterized by the expansion of the inner root sheath epithelium, high expression of the proliferation marker Ki-67, presence of eosinophil granules and the trichohyalin protein in the hyperproliferative cells of the internal root of the hair bulb 193,194. Importantly, TSPyV sT antigen expression has been linked to the deregulation of cellular pathways involved in the control of cell proliferation and apoptosis 195. TSPyV sT and middle T (mT) antigens share the first 196 amino acids, and this region harbors a PP2A-binding motif 196. To date, the role of the mT antigen in the regulation of cell proliferation by TSPyV is unknown; however, the role has been well established for MuPyV 197,198. A recent study has shown that the TSPyV mT antigen interacts with PP2A via a Zn^2+^-binding domain motif and that this interaction is required for the activation of the pro-proliferative MEK/ERK/MNK1 signaling axis 199. As described above, the PP2A-MAPK-regulated pathway is critical for the control of apoptosis and cell proliferation. Therefore, dysregulation of this pathway may lead to uncontrolled cell growth 200,201.

HPyV6, as well as MCPyV, was detected in human skin health samples as part of the microbiome 38,202. Many studies have been conducted to analyze the existence of an association between HPyV6 and lesions of the skin 46,203,204 and other tissues 47,205. To date, these efforts have not led to conclusive results. Interestingly, a recent study detected HPyV6 DNA and the VP1 core protein in samples from patients with melanoma treated with BRAF inhibitors 206. However, and intriguingly, all the samples also tested positive for HPyV7 and HPV. Nonetheless, the HPyV6 DNA load detected suggests that this virus may contribute to epithelial cell proliferation in these patients. The HPyV6 sT antigen contains a PP2A binding domain that may be involved in the activation of MAPK signaling cascade and c-Jun 237.

Some HPyVs have been linked to specific severe pathologies, mainly in immunosuppressed individuals. The growing number of individuals infected with HIV or being treated with immunosuppressant drugs raises the concern that new conditions associated with known or yet-to-be-discovered HPyVs may arise. Therefore, further studies are needed to better characterize these agents and their biology, epidemiology and associations with malignancies in human populations.

## AUTHOR CONTRIBUTIONS

Prado JC prepared the text describing the general characteristics of PyVs, details on Merkel cell PyVs and Table 1. Monezi TA prepared the text describing the general characteristics of TSPyV and its association with human pathologies. She also prepared the illustrations in Figures 1 and 2 and helped prepare Table 1. Amorim AT prepared the text describing the general characteristics of JCPyV and its association with human pathologies. Lino V prepared the text describing the general characteristics of BKPyV and its association with human pathologies. Paladino A revised the entire text and helped prepare Table 1. Boccardo E prepared, revised and corrected the entire text.

## Figures and Tables

**Table 1:** Summary of studies addressing the presence of human polyomaviruses in tumor samples.

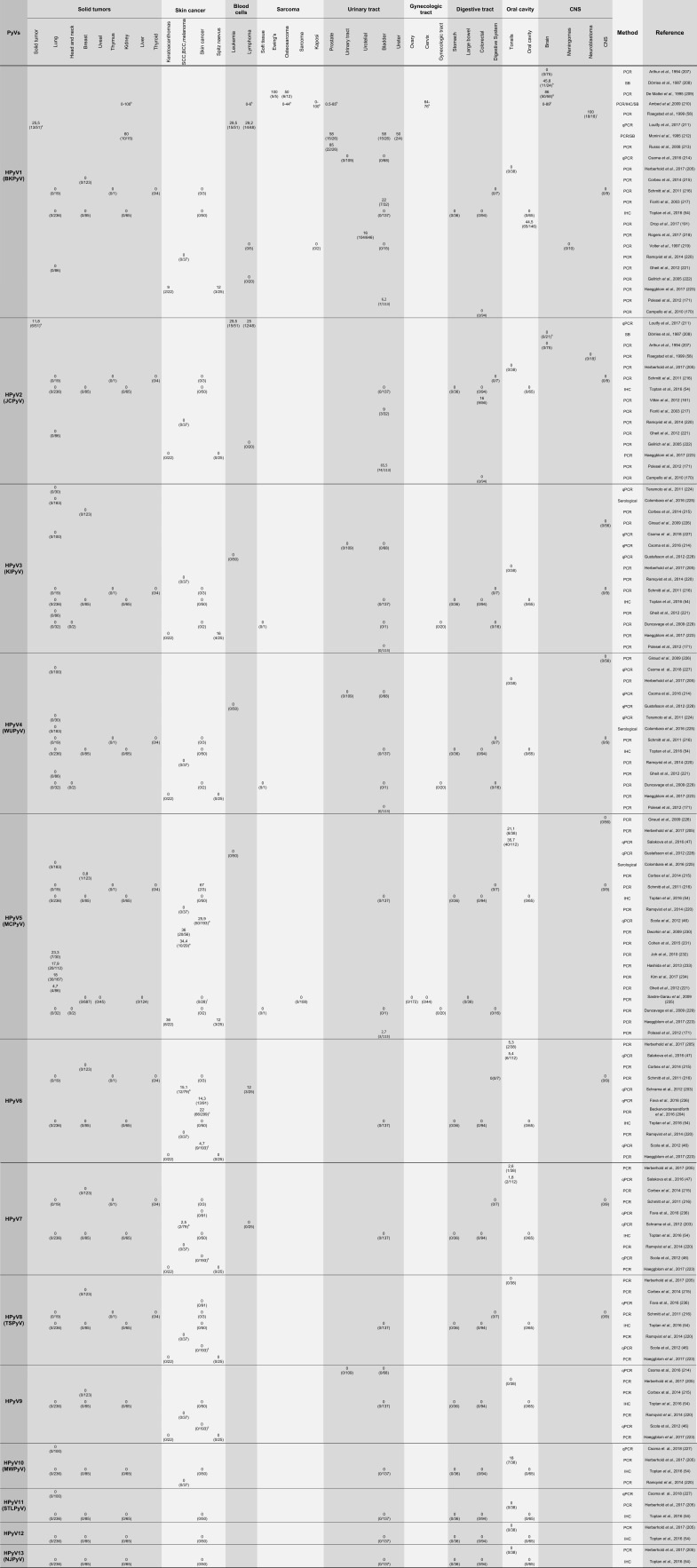

PCR, polymerase chain reaction; qPCR, quantitative polymerase chain reaction; SB, Southern blot hybridization; IHC, immunohistochemistry; IFA, immunofluorescence; ISH, *in situ* hybridization; DDrk, DNA-DNA reassociation kinetics

Sample description and number (when available):

a) (18) Breast; (8) rectal; (7) liver; (3) brain; (3) ovarian; (2) cervical carcinoma; (2) plural mesothelioma; (2) testicular carcinoma; (1) laryngeal carcinoma; (1) bladder; (1) non-small cell lung carcinoma (NSCLC); (1) penis sarcoma; and (2) synovial sarcoma.

b) (21) Squamous cell carcinoma (SCC); (18) basal cell carcinoma (BCC); (20) melanoma; and (20) MCV-neg MCC.

c) (86) SCC; (109) BCC; (45) tricoblastoma; (59) kerathoacanthoma.

d) BCC; SCC; keratoacanthoma; microcystic adnexal carcinoma; atypical fibroxanthoma; facultative SCC precursor lesions; actinic keratosis (AK); and SCC *in situ* (SCCis).

e) Only melanoma tissues from patients treated with serine/threonine-protein kinase B-raf (BRAF) inhibitors.

f) Skin no MCC - BCC, melanoma and other.

g) Tumors from 8 histological types.

h) Tumors from 11 histological types (total n = 24 and 21 for BKPyV and JCPyV, respectively).

i) (13) Primary tumors, (4) post treatment and (1) liver metastasis.

k) Range of prevalence from different studies using serological and molecular assays.
